# Cellular and Network Mechanisms for Temporal Signal Propagation in a Cortical Network Model

**DOI:** 10.3389/fncom.2019.00057

**Published:** 2019-08-27

**Authors:** Zonglu He

**Affiliations:** Faculty of Management and Economics, Kaetsu University, Tokyo, Japan

**Keywords:** nonlinear dynamics, time series modeling, homeostatic encoder, multithreshold decoder, all-or-none modulation, backpropagation, synchronous spiking events, cortical minicolumns

## Abstract

The mechanisms underlying an effective propagation of high intensity information over a background of irregular firing and response latency in cognitive processes remain unclear. Here we propose a SSCCPI circuit to address this issue. We hypothesize that when a high-intensity thalamic input triggers synchronous spike events (SSEs), dense spikes are scattered to many receiving neurons within a cortical column in layer IV, many sparse spike trains are propagated in parallel along minicolumns at a substantially high speed and finally integrated into an output spike train toward or in layer Va. We derive the sufficient conditions for an effective (fast, reliable, and precise) SSCCPI circuit: (i) SSEs are asynchronous (near synchronous); (ii) cortical columns prevent both repeatedly triggering SSEs and incorrectly synaptic connections between adjacent columns; and (iii) the propagator in interneurons is temporally complete fidelity and reliable. We encode the membrane potential responses to stimuli using the non-linear autoregressive integrated process derived by applying Newton's second law to stochastic resilience systems. We introduce a multithreshold decoder to correct encoding errors. Evidence supporting an effective SSCCPI circuit includes that for the condition, (i) time delay enhances SSEs, suggesting that response latency induces SSEs in high-intensity stimuli; irregular firing causes asynchronous SSEs; asynchronous SSEs relate to healthy neurons; and rigorous SSEs relate to brain disorders. For the condition (ii) neurons within a given minicolumn are stereotypically interconnected in the vertical dimension, which prevents repeated triggering SSEs and ensures signal parallel propagation; columnar segregation avoids incorrect synaptic connections between adjacent columns; and signal propagation across layers overwhelmingly prefers columnar direction. For the condition (iii), accumulating experimental evidence supports temporal transfer precision with millisecond fidelity and reliability in interneurons; homeostasis supports a stable fixed-point encoder by regulating changes to synaptic size, synaptic strength, and ion channel function in the membrane; together all-or-none modulation, active backpropagation, additive effects of graded potentials, and response variability functionally support the multithreshold decoder; our simulations demonstrate that the encoder-decoder is temporally complete fidelity and reliable in special intervals contained within the stable fixed-point range. Hence, the SSCCPI circuit provides a possible mechanism of effective signal propagation in cortical networks.

## 1. Introduction

Cortical mechanisms of information propagation in cognitive processes have not been clarified. Neurons convey this information by sending a sequence of action potentials (a spike train) in the brain, but whether this information is embedded in the spike train by rate or temporal coding is a long-debated topic. Although the input–output function of neurons is classically described as the ratios of mean firing rates (Shadlen and Newsome, 1998), the efficiency of rate coding remains controversial (Gautrais and Thorpe, 1998; van Rossum et al., 2002). Accumulating experimental evidence supports temporal precision with millisecond fidelity and reliability (e.g., Gollisch and Meister, 2008). But temporal coding is challenged by neuronal response latency and irregular firing.

Latencies in axonal conduction and synaptic transmission (Uzuntarla et al., 2012) lower transfer speed due to wait times. Both highly variable reliable neurotransmitter releases and the intrinsic fast activation kinetics of interneuronal K^+^ currents can induce highly irregular firing during ongoing, spontaneous activity, and when driven at high firing rates (Stiefel et al., 2013). The irregular firing of cortical neurons may reduce the reliability of spike transmission. That is, temporally effective transfer is seemingly impossible. However, Diesmann et al. (1999) show that precisely synchronized action potentials with millisecond fidelity can propagate within a model of cortical network activity that recapitulates many of the features of biological systems; and Wei and Du (2019) demonstrate that time intervals and periodicity operation can be determined by using an algorithm for simulating a synaptic learning mechanism in a neural circuit model derived from neural-connection structures.

Spiking propagation synchronously through layers is essentially a feed-forward network of neurons (Abeles, 1982a, 1991). Network topology in the feed-forward network determines the propagation of synchronous activity (Guo et al., 2017), suggesting that an optimal network topology relates to synchronous spike events (SSEs) in feed-forward networks. SSEs propagating between groups of neurons in a temporally precise manner through a six-layered, column-arranged neocortex is a hallmark feature of cortical population coding in human and other primate brains. The columnar organization hypothesis is the most widely adopted explanation of cortical information processing. These results suggest that the optimal network topology integrates the functions of SSEs and cortical columns in spiking propagation through the cortex in a feed-forward manner. SSEs occur in various conditions in numerous areas of the cerebral cortex (Abeles, 1982a; Gray et al., 1989). Highly irregular firing is thought only possible from fast, strong dendritic non-linearity or strong SSEs among synaptically connected cells due to inconsistency with the temporal integration of random EPSPs (Softky and Koch, 1993). The temporal sequences of SSEs have been postulated as a working mechanism of activity propagation in the cortex (Abeles and Gerstein, 1988; Diesmann et al., 1999; Ikegaya et al., 2004; Torre et al., 2016a). Neurons within a minicolumn share the same tuning for any given receptive field attribute (Horton and Adams, 2005), while adjacent minicolumns may have different fields (Jones, 2000). Thus, minicolumns may well constitute a fundamental computational unit of the neocortex (Buxhoeveden and Casanova, 2002). Increasing evidence shows that the power of cortical processing is produced by populations of neurons forming dynamic neuronal ensembles (Castejon and Nuñez, 2016). On the other hand, there is an interaction between microscopic and population dynamics (Panzeri et al., 2015). SSEs in temporal encoding depend on single-neuron features (Grewe et al., 2017). Single neuron properties and firing statistics are consistent with physiological data (van Rossum et al., 2002) and the mechanisms of dynamic information storage in cells (Potter et al., 2017).

This study aims to reveal cortical mechanisms that support effective signal propagation over a background of irregular firing and response latencies occurring in cognitive processes. First, we propose the hypothesis of a cortical population circuit from an entry point of rapid transfer of high-intensity signals, incorporating the interneuron encoder-decoder into the cortical population circuit composites a cellular-network model. Then, we derive the conditions for an effective (fast, reliable, and precise) circuit. Finally, we provide evidence from simulations and observations in support of these conditions and hypothesis.

A desirable candidate for action potential encoding should satisfy the following requirements. The neuronal encoder as basic signal processing should be reproducible and reflect the major properties of neurons and circuits in information processing, including inherent non-linearity (Softky and Koch, 1993), ionic homeostasis (Davis and Bezprozvanny, 2001), activity-dependent synaptic dynamics (Fuhrmann et al., 2002), response latency (Uzuntarla et al., 2012), noise (Stiefel et al., 2013), and discreteness (Abbott et al., 2016). Additionally, a spike train is thought to be caused by synaptic stimuli as the bifurcation parameter that triggers a fast transition between quiescent and burst modes by a fixed point and limit cycle (Izhikevich, 2000).

We adopt the non-linear autoregressive integrated (NLARI) process derived by applying Newton's second law to stochastic resilience systems (He, 2007, 2013) in action potential encoding because the model satisfies the above requirements. Moreover, the NALRI's parameter estimation and testing are easy (He, 2014). The dynamics of the cortex have not been thoroughly addressed, although a bifurcation in cortical activity from damped stochastic activity (or a stable fixed point) to high amplitude non-linear oscillations is thought to arise from activity on a limit cycle or chaotic attractor in pathological states such as the onset of a seizure (Deco et al., 2008). The NLARI process can reproduce complete dynamic evolution from a stable to an unstable fixed point and from period cycles to chaos (He, 2018), which prevents missing the possible dynamic mechanisms of neuronal encoding over a wide range of health and disease states.

## 2. Results

### 2.1. SSCCPI Circuit

**Figure 1:**
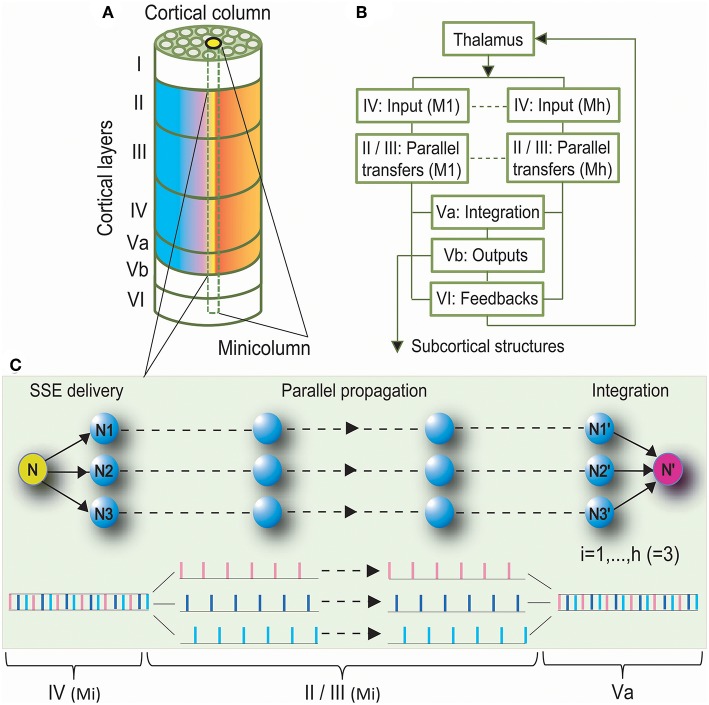
Synchronous Spiking Cortical Column Propagation Integration (SSCCPI) Scheme. **(A)** A cortical organization schematic showing a six-layered, column-arranged neocortex. **(B)** A flowchart showing cortical information processing along the layer IV, → II/III, → Va, and → Vb/VI pathway. **(C)** Schematic illustration of the role of a SSCCPI scheme in response to rapid transfer of high-intensity signals under response latency: a stimulus input from a sensory neuron (N) is delivered by SSEs to neurons (N1–N3) within a cortical column, propagated in parallel to neurons (N1′–N3′), and integrated at a target neuron (N′) for a single-neuron transfer with temporal-complete fidelity.

We proposed the Synchronous Spiking Cortical Column Propagation Integration (SSCCPI) circuit for transfer mechanism in cortical networks (Figure 1). First, we outlined the organization and flowchart of cortical information processing. Figure 1A illustrates an organization schematic of a cortical column through six layers. Neurons in the neocortex are organized vertically into numerous columns with columnar segregation and horizontally in supragranular layers II/III, granular layer IV, and infragranular layers V/VI. Figure 1B illustrates a cortical information processing flowchart. When the activation of sensory receptors scattered throughout peripheral body parts generates a nerve impulse, this sensory input is conveyed via the ascending sensory pathways of the spinal cord and brainstem to the thalamus. The thalamic nuclei relay sensory information to a specific region of the neocortex where it can be processed. Sensory information is thought to be propagated through the cortical column along the layer IV, → II/III, and → V/VI pathway (Buonomano and Merzenich, 1998). Layers II/III interpret sensory signals, decide on the appropriate response, and provide the basis of high-level neural activity in the brain. Layer V projects the main outputs to subcortical structures. Layer VI sends feedback connections to its inputs from the thalamus. Notably, layer V is classically subdivided into sublayers Va and Vb based on the following characteristics (Zilles and Wree, 1995): layers Va and Vb differ dramatically in the morphology of pyramidal cells and their correlation with intrinsic and extrinsic physiological parameters; layer Va pyramidal neurons receive most of their excitatory and inhibitory inputs from intracolumnar sources, especially from layer Va itself, but also from layer IV, and the two layers are the main origin for transcolumnar excitatory inputs. Thus, layer Va may predominantly integrate information intralaminarly as well as from layer IV (Schubert et al., 2006). Hence, we postulated that layer Va integrates thalamic and intracortical inputs from the entire cortical column, while layer Vb projects the main outputs to subcortical structures (see Harris and Mrsic-Flogel, 2013).

A key entry point to addressing our issues was based on the idea that the computational properties of groups of neurons should be an emergent property of the group. Rapid task-related performance or attention to complex sounds induces rapid and adaptive reshaping of retrieve field properties of neurons in accordance with specific behavioral demands and salient sensory cues (Fritz et al., 2003, 2005, 2007; Soto et al., 2006). The rapid and adaptive reshaping of retrieve field properties of neurons could be known as SSEs.

When faced with a behavioral task, sensory neurons need to rapidly elicit high-intensity signals from peripheral nerves to the neocortex, and communication between different areas of the brain substantially increases. A significant increase in the stimulus intensity leads to a high firing rate and high synaptic level. The high firing rate shortens time intervals between consecutive spikes in the spike train, while the high synaptic level lengthens neural response latencies. The shortened spreads of time intervals are far less than the lengthened response latencies, while a spike train is a series of data points indexed in time order. Thus, many spikes in the spike train travel to different neurons from the previous neurons between the last stimulus onset and the beginning of the response. In this manner, many neurons participate in the propagation of the spike train, which is the SSE. SSEs exhibit entire or partial non-overlap-spike delivery (asynchronous SSEs). As the presynaptic discharge rate rises, synaptic depression causes the amplitude of a single postsynaptic current to become inversely proportional to the firing rate. When the presynaptic firing rate exceeds the limiting frequency, the time-averaged postsynaptic current also nearly reaches its saturation value; thus, synaptic connections no longer convey information about the presynaptic discharge rate (Gerstner et al., 1997). That is, once the increased stimulus intensity exceeds the ability of individual neurons to process information, spikes in the spike train have to be delivered synchronously into many neurons. Then, SSEs with entire overlap-spike delivery (rigorous SSEs) occur. The same spike is received in parallel by massive individual neurons. Notably, irregular synaptic inputs makes SSE delivery at least partially non-overlapping. Thus, these individual neurons usually have lower synaptic input levels, which shortens their response latencies. The transfer of a spike train with low density has a shorter latency than that of a spike train with high density; thus, SSEs were effective in enhancing transfer speed by avoiding the lengthened response latency caused by high-intensity signals. Consequently, we assumed that SSEs were a consequence of cortical population responses to rapid transfer of high-intensity signals with neural response latency.

The question then arises as to which mechanism would ensure information fidelity during disassembly signal propagation such that the integration spike train of decomposed spike trains can return to the original spike train. Asynchronous SSEs perform the conversion of a spike train with high density into many spike trains with low density such that intervals between spikes usually become greater than response latency. Accordingly, there is no reason to repeatedly activate SSEs in response to high-intensity signals. After one SSE delivery, those spike trains with low density should be propagated through layers II/III first and then integrated into an output spike train as a convergent input from simultaneously spiking neurons onto a target neuron in layer Va as previously suggested by Diesmann et al. (1999). If the output spike train returns to the thalamic input, the disassembly propagation of high-intensity signals is successful. There are two prerequisites for returning to the initial input. A prerequisite is that SSE delivery is not repeatedly activated; otherwise, these separately propagated spikes disperse and eventually die out. A further prerequisite is that disassembled spikes do not bump into other neurons with different receptive fields during parallel propagation; otherwise, incorrect synaptic connections result in information loss and distortion. Cortical columns (Mountcastle et al., 1955) in the vertical dimension and columnar segregation could meet these two prerequisites. Neurons within a given minicolumn were stereotypically interconnected in the vertical dimension, which prevented repeated activation of SSEs and ensured parallel propagation of spike trains, while columnar segregation could prevent incorrect synaptic connections between adjacent columns. When the stimulus intensity exceeds the ability of individual minicolumns to process information, the stimulus spikes have to be delivered synchronously into multiple minicolumns within a given macrocolumn.

We summarized that rapid transfer of high-intensity signals could be achieved by the following components: SSEs delivering a high-intensity thalamic stimulus input to many neurons within a cortical column as many spike trains with low density in layer IV, parallel propagation of these spike trains with low density along minicolumns through layers II/III, and integrating these spike trains into an output toward or in layer Va. Parallel propagation of many sparse spike trains through SSE delivery enhances the transfer speed of high-intensity signals, while vertical columns with segregation ensure parallel-propagation fidelity. A circuit with these three components is called the SSCCPI Circuit. Figure 1C is a simplified SSCCPI circuit. Formally, we have the following definition:

*Definition 1*. The Synchronous Spiking Cortical Column Propagation Integration (SSCCPI) circuit is a neural circuit by which SSEs deliver dense spikes of a thalamic high-intensity stimulus input into many receiving neurons within a cortical column in layer IV first. Then, many sparse spike trains are propagated in parallel by a propagator along cortical minicolumns through layers II/III and finally integrated into an output spike train toward or in layer Va.

### 2.2. Effective SSCCPI Conditions

#### 2.2.1. Coding Strategy

**Figure 2:**
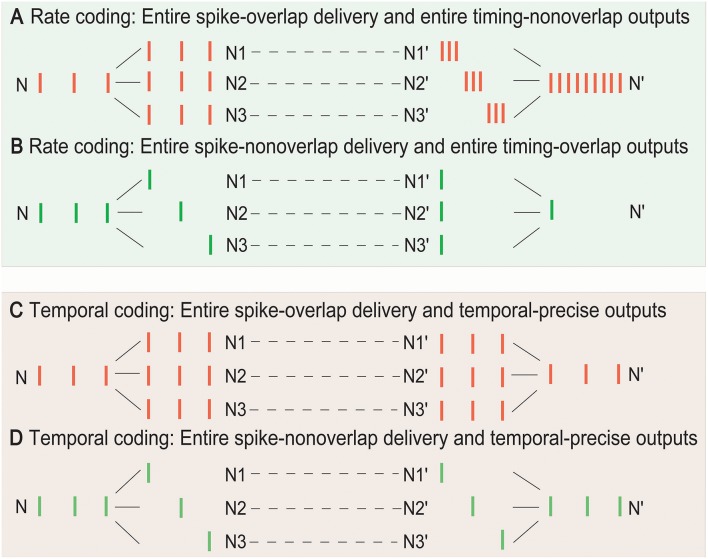
Temporal and rate coding in SSCCPI transfer precision. Consider single-neuron transfer with complete fidelity. **(A)** Entire spike-overlap delivery of SSEs to N1–N3 and entire timing-non-overlap outputs in N1′–N3′ in rate coding. **(B)** Entire spike-non-overlap delivery of SSEs to N1–N3 and entire timing-overlap outputs in N1′–N3′ in rate coding. **(C)** Entire spike-overlap delivery of SSEs to N1–N3 and temporal-precise outputs in N1′–N3′ in temporal coding. **(D)** Entire spike-nonoverlap delivery of SSEs to N1–N3 and temporal-precise outputs in N1′–N3′ in temporal coding.

Determining whether the rate or temporal coding is more suitable for the SSCCPI circuit in different overlapping degrees of SSE delivery is important. Without loss of generality, we focused on entire overlap deliveries and entire non-overlap deliveries in single-neuron signal transfer with complete fidelity. Figure 2 showed the performance comparison between these two coding strategies.

In rate coding, when neurons within a cortical column received and propagated the same input spike train (SSEs with entire spike-overlap delivery), the output firing rate after integration could significantly increase up to *h*-fold greater than the input firing rate if the output spike timing was entirely non-overlapping where *h* was the number of the receiving neurons, although the firing rate before, and after propagation remained unchanged (Figure 2A). When neurons within a cortical column received completely different input spike trains (SSEs with entire spike-non-overlap delivery), the output firing rate could significantly decrease if the output spike timing was entirely overlapping (Figure 2B). Thus, the rate coding could not exactly reflect SSCCPI transfer precision. In contrast, in temporal coding, the output spike train could return to the initial input spike train, regardless of SSEs with entire spike-overlap delivery (Figure 2C) or entire spike-non-overlap delivery (Figure 2D). Therefore, the SSCCPI circuit should utilize temporal coding because temporal coding makes it possible to maintain SSCCPI transfer precision with complete fidelity (Figures 2C,D). In temporal coding, SSEs were effective in enhancing transfer speed for partial or entire spike-non-overlap delivery but ineffective for entire spike-overlap delivery.

#### 2.2.2. Factors Influencing Precision

The symbol error rate is an indicator of signal propagation efficiency in data communications. To distinguish neuronal communications from the data communications, we introduced the propagation success rate to assess the propagation precision of nerve signals. Consider an all-or-none modulation as the conversion rule of a raw input in layer IV and a final output of neurons in layer Va in the firing of neurons. The all-or-none modulation is a principle that the strength of a response of a neuron to a stimulus is not dependent upon the strength of the stimulus whereby the neuron gives a complete response if the stimulus exceeds the threshold potential; otherwise, there is no response. For these reasons, we introduced the propagation success rate as follows:

*Definition 2*. (i) The propagation success rate of a spike train with *n* points in single neurons through *m* relays is defined by:

(1) $$\begin{array}{l} r\left(m,n\right)=\left[1-\frac{{\left({\text{v}}_{m}-{\text{v}}_{0}\right)}^{⊺}\left({\text{v}}_{m}-{\text{v}}_{0}\right)}{n}\right]\times 100\% \end{array}$$

with

(2) $$v_{0,t}=\{\begin{array}{c} 1\\ 0 \end{array}|\begin{array}{c} \text{if }\varepsilon_{t}\geq c_{3}\\ \text{if }\varepsilon_{t}<c_{3} \end{array},v_{m,t}=\{\begin{array}{c} 1\\ 0 \end{array}|\begin{array}{c} \text{if }Y_{m,t}\geq c_{3}\\ \text{if }Y_{m,t}<c_{3} \end{array}$$

where ε_*t*_ is a raw input stimulus, *v*_0,*t*_ is the initial received input, *v*_*m,t*_ is the final output of a target neuron at time *t*, and ${\text{v}}_{i}={\left(v_{i,1},\cdot \cdot \cdot \;,v_{i,n}\right)}^{⊺}$ for *i* = 0, *m*.

(ii) The signal transfer has complete fidelity or success if *r*(*m, n*) is ~100% or *v*_*m,t*_ = *v*_0,*t*_ for almost *t* and the signal transfer is a complete distortion or failure if *r*(*m, n*) is ~0% or *v*_*m,t*_ ≠ *v*_0,*t*_ for almost *t*.

**Figure 3:**
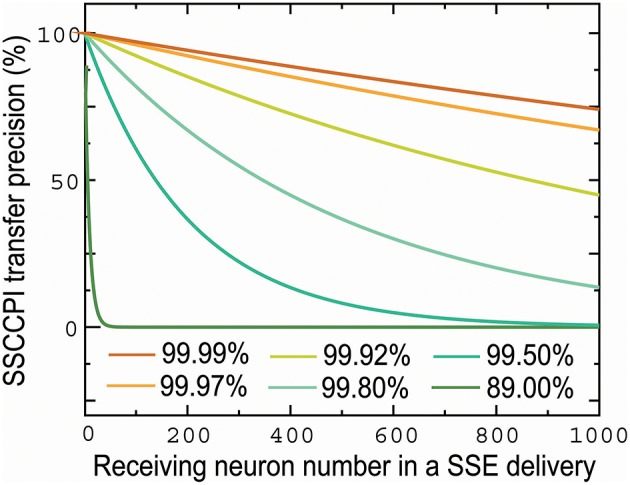
Influences of the number of receiving neurons and single-neuron transfer precision on SSCCPI transfer precision. SSCCPI transfer precision decays exponentially with an increasing number of receiving neurons from 1 to 1,000 in SSE delivery and significantly with a decreasing single-neuron transfer precision from 99.99 to 89.00%.

The propagation success rate in the SSCCPI circuit can be given by *r*^*h*^(*m, n*) where *h* is the number of receiving neurons in a cortical minicolumn. It is normal for each neuron to have 1, 000 connections. Figure 3 shows that the SSCCPI propagation success rate decreases distinctly exponentially with an increasing receiving neuron number when the propagation success rate in single neurons is lower than 99.92%. For example, the SSCCPI propagation success rates in 1,000 receiving neurons are 90.48, 74.08, and 44.92% when the single-neuron propagation success rates are 99.99, 99.97, and 99.92%, respectively. Consider minicolumns with 80–120 neurons. The SSCCPI propagation success rate in 80 receiving neurons is almost zero for the single-neuron propagation success rate 89.00%. These results imply that the faster SSCCPI transfer requires the higher interneuron transfer precision; while a rapid SSCCPI transfer certainly results from complete fidelity transfer of temporal information in interneurons. This result shows that parallel communication requires far higher transfer precision per line than serial communication in critical networks.

**Figure 4:**
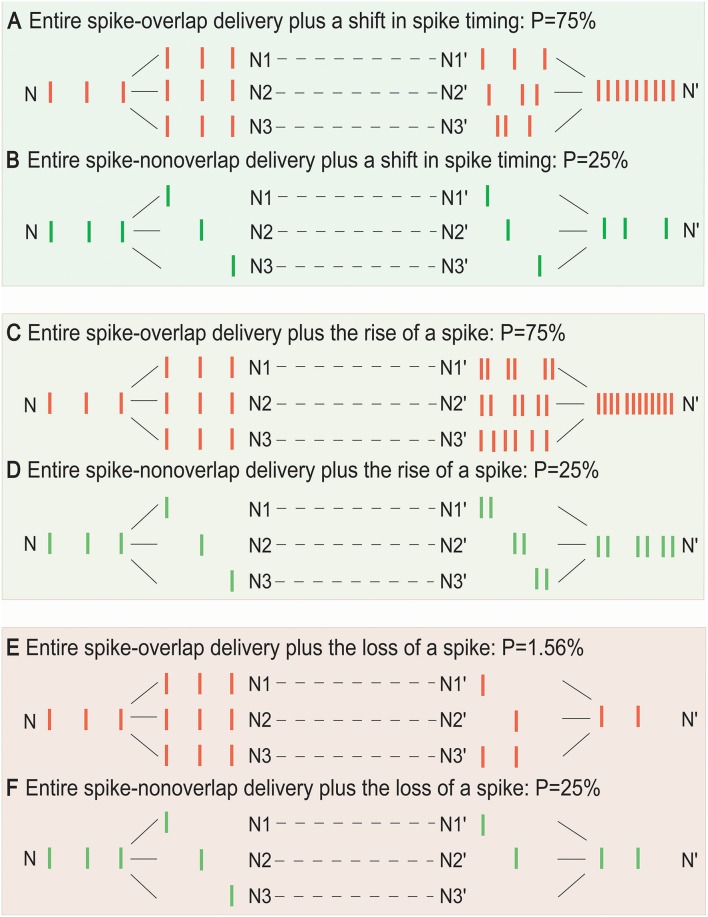
Influence of the overlap degree of SSE delivery and single-neuron transfer mistakes on SSCCPI transfer precision. For the entire spike-overlap delivery of SSEs, the largest output firing rate can be up to *h*-fold greater than the input firing rate with probability *hmp*_*s*_ **(A)**, *h* + 1-fold greater than the input firing rate with probability *hmp*_*i*_ **(C)**, and is less than the input firing rate with probability ${\left(mp_{d}\right)}^{h}$ **(E)**. For the entire spike-non-overlap delivery of SSEs, the largest output firing rate can be the same as the input firing rate with probability *mp*_*s*_ **(B)**, be up to 2-fold greater than the input firing rate with probability *mp*_*i*_ **(D)**, and less than the input firing rate with probability *mp*_*d*_ **(F)** for *m* relays, *h* receiving neuron number, and *p*_*s*_, *p*_*i*_, *p*_*d*_ probabilities of the occurrence of a shift in spike timing, the rise of a spike, and the loss of a spike in single neurons.

Furthermore, Figure 4 demonstrated that the SSCCPI propagation precision could vary with the overlapping degree of SSE delivery plus transfer mistake types in single neurons (or interneurons). When a spike that results in an action potential passes through a neuron, transfer mistakes in single neurons result from a shift in spike timing, the rise of a spike, and the loss of a spike. We considered the SSCCPI circuit with *m* relays and *h* receiving neurons and *p*_*s*_, *p*_*i*_, and *p*_*d*_ as the probabilities for the occurrence of a shift in spike timing, the rise of a spike, and the loss of a spike, respectively. Without loss of generality, let *p*_*s*_ = *p*_*i*_ = *p*_*d*_ = *p* (*p* = 5%, *h* = 3, and *m* = 5 for Figure 4). For the entire spike-overlap delivery of SSEs, the largest output firing rate can be up to *h*-fold (Figure 4A) or *h* + 1-fold greater than the input firing rate with probability *hmp* (Figure 4C) because of the transfer mistakes in a shift in spike timing or the rise of a spike, and the output firing rate can become less than the input firing rate with probability (*mp*)^*h*^ (Figure 4E) because of the transfer mistake in the loss of a spike. For the entire spike-non-overlap delivery of SSEs, the output firing rate can remain unchanged (Figure 4B) or become greater (Figure 4D) or less than the input firing rate with probability *mp* (Figure 4F) because of the transfer mistake in a shift in spike timing, the rise of a spike or the loss of a spike. The number of the receiving neurons is usually relatively large. Thus, substantially increased output firing rate could result only from rigorous SSEs plus the interneuron transfer mistake in a shift in spike timing or the rise of a spike, while any change in the output spike train was unlikely to result from asynchronous SSEs plus any interneuron transfer mistakes.

#### 2.2.3. Factors Influencing Speed

To explore what affects the SSCCPI' propagation speed, we gave the following

*Definition 3*. The speed of signal propagation in neural networks means the speed of travel of a given nerve signal (a spike train as a unit signal) from one place to another in the brain.

The propagation time of signals in neural networks is related to both distances of space and signal intensity. The processing time of an impulse contains time spent on impulse axonal propagation and synaptic transmission. Usually, the processing time of an impulse remains unchanged for an individual neuron. Hence, the propagation time varies primarily with distances of space when signal intensity is not sufficient to trigger a SSE delivery. When high signal intensity triggers a SSE delivery, the propagation time relates not only to distances of space, but also depends crucially on the spike density of those spike trains segregated by SSE delivery. Consider that neurons are able to autochthonously select the shortest pathway in sending the signal to target neurons, that is, the space potential to improving the transfer speed is less. Thus, a great potential for improving the propagation speed relies crucially on shortening the waiting time to process the signal.

**Figure 5:**
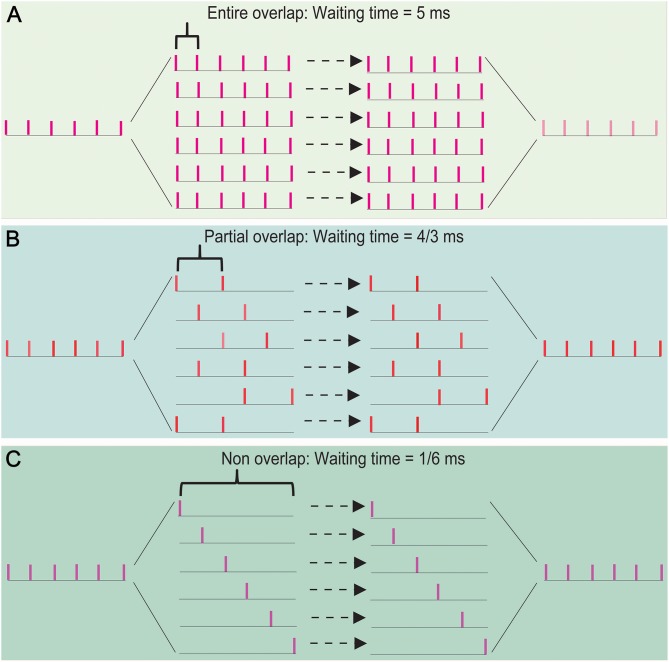
The effects of overlapping degree of spike trains on the propagation speed in a SSE delivery. The waiting time takes 5 ms for an entire spike-overlapping case **(A)**, 4/3 ms for a partial spike-overlapping case **(B)**, and 1/6 ms for a non spike-overlapping case **(C)**. This shows that a lower spike-overlap of a SSE delivery has the shorter waiting time, namely the faster transfer speed.

Let us consider the case: (i) the processing time for a spike per neuron is 1 ms (usually, the absolute refractory period takes about 1–2 ms), (ii) a thalamic spike train has *p* spikes and 1/*p* ms equal interval, and (iii) the least interspike interval in these spike trains is *q*/*p* ms (equivalent to the least interspike interval is enlarged *q* times) for 1 ≤ *q* ≤ *p* − 1. The number of spikes in each spike train is not greater than *p*/*q*. The waiting time of a spike is not >1 − *q*/*p* (ms). Thus, the sum of the waiting time of these spike trains is not >(*p*/*q*) × (1 − *q*/*p*) = *p*/*q* − 1 (ms). Therefore, the waiting time can be shortened about *q* times by a SSE delivery. Figure 5 shows the cases (*p, q*) is given by (6, 1) **(A)**, (6, 2) **(B)**, and (6, 5) **(C)**. Their waiting times are 5, 4/3, and 1/6 (ms), which are not >*p*/*q* − 1 (ms).

The above results showed that the waiting time of signal processing depends primarily on the degree of overlap of spike trains segregated by a thalamic spike train. Thus, the propagation speed could be significantly improved by SSE delivery; a great potential for improving the propagation speed could be realized by increasing the degree of spike-non-overlap of spike trains in interneurons.

#### 2.2.4. Factors Influencing Reliability

Whether the SSCCPI transfer precision remains reliable over a background of irregular synaptic inputs warrants investigation. Hence, it is necessary to define the reliability of signal transfer.

*Definition 4*. A cortical circuit has a reliable transfer function if the transfer precision of temporal information in the neocortex is not influenced by the input irregularity.

Under the normal states of SSE delivery and cortical columns for a given overlapping degree, the SSCCPI propagation precision depends on whether its single-neuron (or interneuron) transfer has complete fidelity in a temporally precise manner according to the results in the above section. In other words, if the complete fidelity of a single-neuron (or interneuron) transfer is reliable, then the SSCCPI transfer precision is reliable. Accordingly, SSCCPI transfer reliability is reducible to the reliability of the single-neuron transfer.

#### 2.2.5. Neuronal Encoder

The NLARI process with κ_1_ = 1 can be specified by:

(3) $$\begin{array}{l} X_{t}=\theta_{0}+\left(1+\theta_{1}\right)X_{t-1}-\theta_{1}X_{t\;-2}\;+\;\theta_{2}\frac{-\left(X_{t-\kappa_{2}}-\mu_{t-\kappa_{2}}\right)}{\exp\left({\left(X_{t-\;\kappa_{2}}-\mu_{t-\kappa_{2}}\right)}^{2}\right)}+\varepsilon_{t} \end{array}$$

where, θ_0_ = ω, θ_1_ = 1 − α, θ_2_ = β, *X*_*t* −*j*_ lags *X*_*t*_ by *j* steps for *j* = 1, ··· , κ_2_; μ_*t*_ = *E*(*X*_*t*_ ∣ *X*_0_, *X*_−1_) = *X*_0_ + (ω/α)*t* if ε_*t*_ = ϵ_*t*_ − *E*(ϵ_*t*_) is Gaussian noise. ϵ_*t*_ is external disturbance with mean ω = *E*(ϵ_*t*_) and variance $\sigma^{2}=var\left(\epsilon_{t}\right)$ at time *t*. α is the resistance coefficient, β is the restoration coefficient, γ = β/(4 − 2α) is the stability coefficient, and κ_1_ and κ_2_ are time lags in resistance and restoration (He, 2007, 2013). In the absence of a restoring force (β = 0), Equation (1) is a non-stationary unit root process far from equilibrium (He, 2007). In a lack of background disturbance (σ = 0), Equation (1) is the deterministic system with a fixed point and a two-period cycle ${\left(-1\right)}^{t}\sqrt{\ln\;\gamma }$ for non-null initial values in κ_1_ = κ_2_ = 1 (He, 2013). The fixed point is exponentially asymptotically stable if γ ∈ (0, 1), while the periodic cycle is exponentially asymptotically stable if $\gamma \in \left(1,\sqrt{e}\right)$. Equation (1) represents unstable period cycles if γ ∈ (1, 3.07) and chaos if γ ∈ (3.07, + ∞) (He, 2018). The fixed point may describe the dynamic mechanism of ionic homeostasis, while together the fixed point and periodic cycle may produce transitions between resting and spiking states.

A dynamic system can be described by the NLARI process if the system sustains an external force, which may cause a deviation from equilibrium (mean), resistance that prevents fast changes, and a restoration force that returns the perturbed system to its mean by a pair of opposite components (He, 2018). For this reason, we focused on exploring whether the membrane potential in response to synaptic stimulus sustains the above-mentioned three forces.

The membrane potential in response to synaptic stimulus is primarily achieved through the difference in membrane permeability to K^+^ ions and Na^+^ ions. At rest, all Na^+^ channels and most K^+^ channels are closed, and the Na^+^–K^+^ transporter pumps K^+^ ions into the neuron and Na^+^ ions out, creating a net electrochemical force driving Na ^+^ into the neuron. A synaptic stimulus causes some Na^+^ channels of a neuron to open, allowing Na^+^ ions to enter the neuron. The net electrochemical force driving Na^+^ into the neuron causes the membrane to depolarize. If the threshold of excitation is reached, all the Na^+^ channels open. At the peak action potential, Na^+^ channels close while K^+^ channels open, allowing K^+^ ions to leave the neuron. The membrane starts to repolarize through a net electrochemical force driving K^+^ out of the neuron and becomes hyperpolarized when more K^+^ ions are on the outside than Na^+^ ions are on the inside. During a refractory period, the Na^+^–K^+^ pump moves Na^+^ ions to the outside and K^+^ ions to the inside using energy from the hydrolysis of ATP against the net electrochemical gradients of both ions. The Na^+^ and K^+^ distributions are restored to the resting state, and a net electrochemical force driving Na^+^ into the neuron brings the membrane back to the resting state.

In summary, excitatory and inhibitory synaptic input (ϵ_*t*_) with mean ω and variance σ^2^ drives the membrane potential (*X*_*t*_) away from the resting potential (*V*_*rest*_ = μ_*t*_), which may cause depolarization. A net electrochemical force driving Na^+^ ion influx or K^+^ ion efflux causes depolarization or repolarization of the neuron, while the Na^+^–K^+^ pump derives Na^+^ out and K^+^ into the neuron to return to the ionic distribution across the membrane at rest. Thus, the net electrochemical force and the Na^+^–K^+^ pump provide a restoration force that maintains homeostasis by returning the perturbed membrane potential to the resting potential. Finally, the plasma membrane provides high resistance that impedes the movement of charges across it, which hinders rapid changes in its potential. Thus, the neuronal response sustains the three required forces. The action potential occurs only at nodes of Ranvier with unmyelinated axons such that the nerve signal appears to jump from node to node and at the trigger zone if an excitatory local potential arrives and remains strong enough to open channels and generate an action potential. Ionic homeostasis is maintained through the regulation of the levels of voltage-gated channels, densities of neurotransmitter receptors, and synapse numbers and strength (Davis and Bezprozvanny, 2001; Dubyak, 2004). Hence, the NLARI process can be used to encode cellular and axonal propagation. Synaptic latency is ~1 ms. We may wish to consider latencies in the membrane resistance and restoration κ_1_ = κ_2_ = 1. Let *Y*_*t*_ = *X*_*t*_ − μ_*t*_. Due to μ_*t*_ = *X*_0_ − (ω/α)*t* for Gaussian noise (He, 2018), *Y*_*t*_ = *X*_*t*_ − *V*_*rest*_ = *X*_*t*_ − *X*_0_ − (ω/α)*t*. Then, Equation (3) can be rewritten as:

(4) $$\begin{array}{l} Y_{t}=\left(2-\alpha \right)Y_{t\;-1}-\left(1-\alpha \right)Y_{t\;-2}+\beta \frac{-Y_{t\;-1}}{\exp\left(Y_{t\;-1}^{2}\right)}+\varepsilon_{t} \end{array}$$

which describes the membrane potential variability driven by background synaptic input ϵ_*t*_ = ε_*t*_ + ω with mean ω and variance σ^2^. α is the membrane electrical resistance coefficient that reflects the electrical resistivity of the opposing flows across the membrane for a given electrical potential, depending on the number, and permeability of channels to Na^+^, K^+^, Ca^2+^, and Cl^−^. β is the membrane potential restoration coefficient that reflects the strength of restoring force to return the resting potential, depending on the magnitude of a net electrochemical force driving Na^+^ and K^+^ ion influx/efflux across the membrane and synaptic plasticity of strengthening/weakening between neighboring synapses in response to increases and decreases in their activity, and reduced ATP availability lowers the membrane potential restoration coefficient because ATP shortage disrupts K^+^/Na^+^ homeostasis resulting in a chronic depolarization (Le Masson et al., 2014). γ is the membrane stability coefficient, and κ_1_ and κ_2_ are response delays in the membrane electrical resistance and membrane potential restoration. If γ ∈ (0, 1), Equation (4) represents a homeostatic encoder with a stable fixed point.

**Figure 6:**
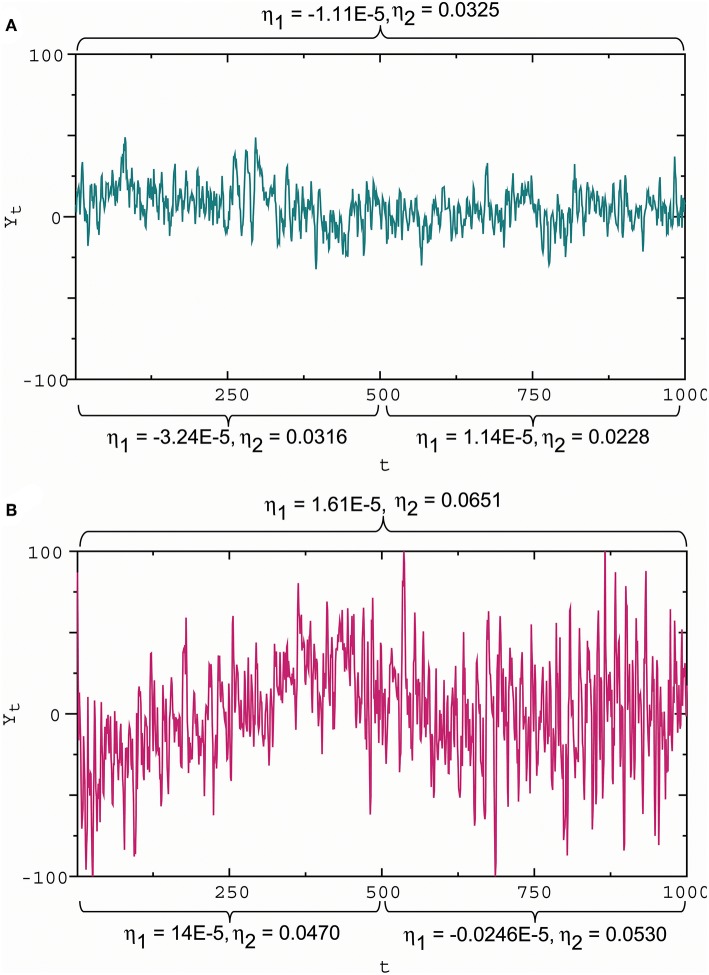
Slope and amplitude of fluctuations and the waveform indicators. **(A)** Small amplitude fluctuations with a gentle downward slope in the first half and almost zero slope in the second half, corresponding to the slope indicator $\eta_{1}=-1.11\times 10^{-5}$ and the amplitude indicator η_2_ = 0.0325 for the whole interval, $\eta_{1}=-3.24\times 10^{-5}$ and η_2_ = 0.0316 for the first half, and $\eta_{1}=1.14\times 10^{-5}$ and η_2_ = 0.0228 for the second half. **(B)** Large amplitude fluctuations with a steep upward slope in the first half and a gentle downward slope in the second half, corresponding to $\eta_{1}=1.61\times 10^{-5}$ and η_2_ = 0.0651 for the whole interval, $\eta_{1}=14\times 10^{-5}$ and η_2_ = 0.0470 for the first half, and $\eta_{1}=-0.0246\times 10^{-5}$ and η_2_ = 0.0530 for the second half. These results show that a positive/negative slope indicator reflects an upward/downward slope, while a large/small absolute slope indicator describes a steep/gentle slope; a large/small amplitude indicator reflects a high/low amplitude. Here these wave indicator values were the estimates based on observational data.

To assess the influence of synaptic stimuli on the membrane potential pattern, we considered the wave indicators developed by He (2018). For Gaussian noise, the ratio η_1_ = ω/α represents the slope of the mean line provided by *E*(*X*_*t*_ ∣ *X*_0_, *X*_−1_) = *X*_0_ + (ω/α)*t*. Thus, the ratio can be viewed as a slope indicator. Moreover, the ratio η_2_ = σ/β is strongly positively correlated with the standard deviation of the data generated by Equation (4) (for details see He, 2018), while the standard derivation of disturbances is a measure of how far the signal fluctuates from the mean. For this reason, the σ/β ratio can be viewed as a wave amplitude indicator. From Figure 6, we see the capability of the wave indicators to measure the slope and amplitude of fluctuations: the slope and amplitude indicator values in Figure 6B are 1.45 times and 2 times the slope and amplitude indicator values in Figure 6A, which is consistent with large and small amplitude fluctuations with steep and gentle slopes (Figures 6A,B), respectively. Sometimes the wave indicators have better performance in the subdivided observation intervals. For example, consider the whole interval subdivided into the two intervals. For the first half, the slope and amplitude indicator values in Figure 6B are 4.32 times and 1.49 times the slope and amplitude indicator values in Figure 6A. The measured results in the subdivided intervals are closer to actual fluctuation patterns than the whole interval. For these reasons, we introduced the membrane potential waveform indicators below:

*Definition 5*. The membrane potential slope indicator is given by:

(5) $$\begin{array}{l} \eta_{1}=\frac{\omega }{\alpha } \end{array}$$

and the membrane potential amplitude indicator is given by:

(6) $$\begin{array}{l} \eta_{2}=\frac{\sigma }{\beta } \end{array}$$

where, α is the membrane electrical resistance coefficient, β is the membrane potential restoration coefficient, ω is the mean of synaptic stimulus input, and σ is the standard derivation of synaptic stimulus input.

According to the definition of the membrane potential waveform indicators, the relative strength of the membrane electrical resistance and membrane potential restoring force to synaptic input determines the scale of membrane potential fluctuations in slope and amplitude. The standard deviation is a measure of how far the signal fluctuates from the mean, but one cannot extract more information than controls the amplitude of fluctuations. Furthermore, the waveform indicators are the membrane potential fractal indicators (He, 2018).

#### 2.2.6. Propagator With Encoder

Consider a propagator for a simple reflex circuit. A reflex circuit can be as simple as a single synapse located between sensory and motor neurons. The SSCCPI circuit is a feed-forward network of neurons with multiple layers. Thus, each neuron in the receiving layer is excited by neurons in the previous layer. In this case, the response of the last neuron to a received stimulus could be regarded as the incoming stimulus into the connected neuron. By incorporating the incoming stimulus into (Equation 4), we obtained a propagator with an encoder in nomodulation. Considering a ll-or-none modulation as the regulation of the incoming stimulus, we could obtain a propagator with an encoder in all-or-none modulation. Formally, we introduced the propagators:

*Definition 6*. Consider a spike train with *n* points as the combination of spikes and silences through *m* relays. The propagator with an encoder is given by:

(7) $$\begin{array}{l} Y_{i,t}=\left(2-\alpha \right)Y_{i,t-1}-\left(1-\alpha \right)Y_{i,t\;-2}+\beta \frac{-Y_{i,t-1}}{\exp\left(Y_{i,t\;-1}^{2}\right)}+Y_{i\;-1,\;t} \end{array}$$

where, *Y*_*i,t*_ represents the response of the *i*th interneuron to the *t* th stimulus *Y*_*i* −1, *t*_ at time *t* for nomodulation and *Y*_*i* −1, *t*_ = *c*_1_ if *Y*_*i* −1, *t*_ ≥ *c*_1_ and $Y_{i\;-1,t}=v_{t}^{\left(1\right)}$ with $v_{t}^{\left(1\right)}\sim i.i.d.N\left(0,\sigma_{1}^{2}\right)$ if *Y*_*i* −1, *t*_ < *c*_1_ for *i* ≥ 2 for all-or-none modulation; *c*_1_ is a threshold value; and initial values *Y*_0,*t*_ = ε_*t*_, *Y*_*i*, −1_ = *Y*_*i*, 0_ = 0, and ε_*t*_ represents the initial received stimulus at time *t* for *i* = 1, ··· , *m* and *t* = 1, ··· , *n*.

Note that for all-or-none modulation, *Y*_*i* −1, *t*_ = 0 if *Y*_*i* −1, *t*_ < *c*_1_, but we let $Y_{i\;-1,t}=v_{t}^{\left(1\right)}$ in order to represent intrinsic noise and extrinsic or synaptic noise with a small variance.

#### 2.2.7. Propagator With Encoder–Decoder

**Figure 7:**
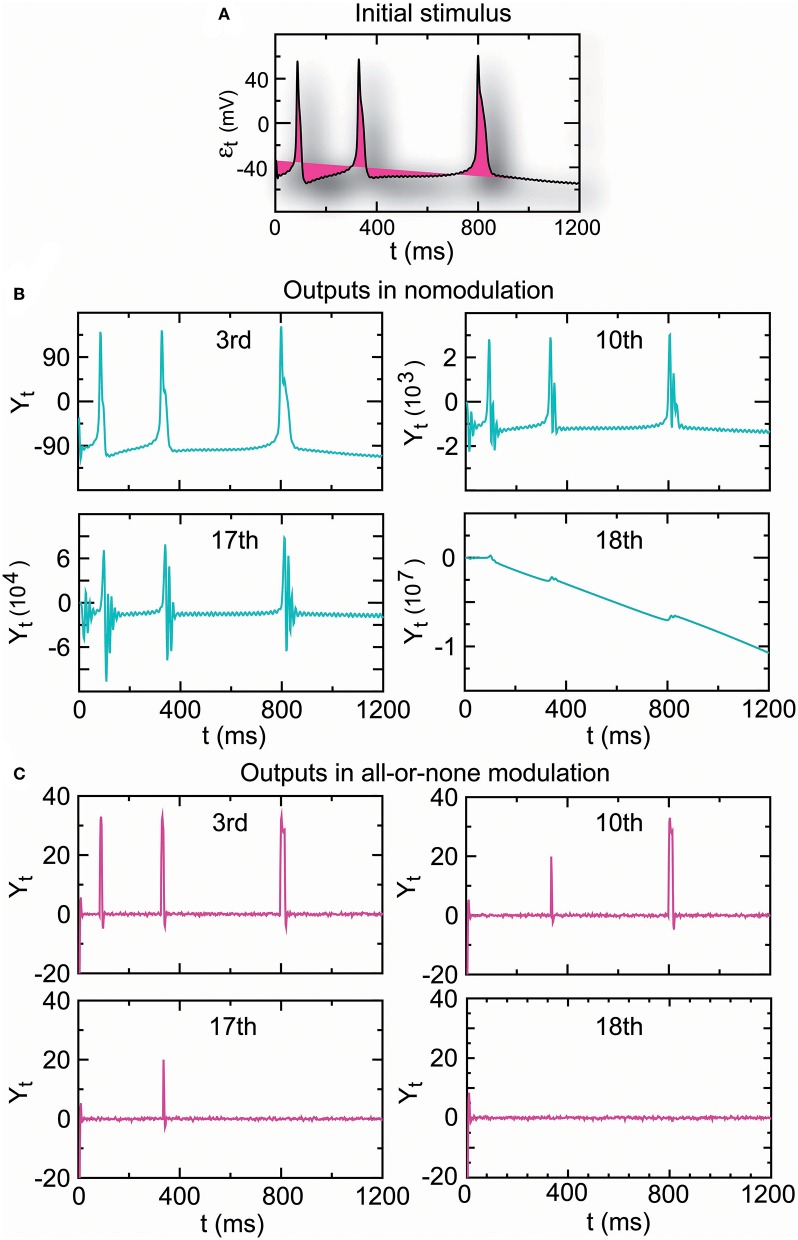
The amplitude of the output spike train driven by real stimuli **(A)** is significantly increased in nomodulation **(B)** and decreased in all-or-none modulation **(C)** by repeatedly running the homeostatic encoder. These results indicate that all-or-none modulation prevents an over response but fails to avoid an under response, which can result in information loss when the encoder is executed repeatedly.

A complex reflex circuit possesses the integration center in the cerebrum, spinal cord, or brainstem where conscious thoughts are initiated. Ascending sensory neurons and descending upper motor neurons (relay interneurons) function as sensory and motor connections and assist in the integration and interpretation of data. The responses of a group of neurons to a stimulus have errors. Whether the accumulated response errors after many relays induce signal loss warrants investigation. Thus, we examined the encoding errors when the propagator with an encoder in Equation (7) for nomodulation and all-or-none modulation (see Definition 6) were operated for *m* times. As the iteration number increased, the initial stimulus of real spike trains (Figure 7A) became significantly enlarged for nomodulation (Figure 7B) and was attenuated for all-or-none modulation (Figure 7C). The simulation result that nerve impulses are significantly enlarged in the firing of a neuron (Figure 7B) reflects the phenomenon that the opening of voltage-gated channels in the course of an action potential produces typically significantly larger currents than the initial stimulating current. Fortunately, all-or-none modulation as a neural regulation of ultra response to stimulus avoids the significant enlargement of impulses (Figure 7C). Although all-or-none modulation prevents an over response, it fails to avoid an under response (Figure 7C). Interestingly, no evidence that all-or-none modulation fails to avoid an under response suggests the existence of a hidden mechanism by which the under response is modulated by supplementing the attenuated currents. In fact, currents produced by the opening of voltage-gated channels are typically larger than the current of the original stimulus, while a voltage stimulus decays exponentially relative to the distance from the synapse and with neurotransmitter binding time. The two opposite tendencies suggest the existence of a back-propagating action potential under the homeostatic regulation to avoid the under response. Based on these reasons, we developed the following decoder for correcting errors caused by the encoder in Equation (7).

*Definition 7*. The multithreshold decoder to correct response errors is given by:

(8) $$\varepsilon_{i,t}=\{\begin{array}{l} c_{1}\\ Y_{i-1,t}\;\\ c_{2}\\ v_{t}^{\left(1\right)} \end{array}|\begin{array}{l} \text{if }Y_{i\;-1,t}\geq c_{1}\;\\ \text{if }c_{2}\leq Y_{i\;-1,t}<c_{1}\\ \text{if }c_{3}\leq Y_{i\;-1,t}<c_{2}\\ \text{if }Y_{i\;-1,t}<c_{3} \end{array}$$

which is the incoming stimulus entering the next neuron in the following encoder:

(9) $$\begin{array}{l} Y_{i,t}=\left(2-\alpha \right)Y_{i,t-1}-\left(1-\alpha \right)Y_{i,t-2}+\beta \frac{-Y_{i,t-1}}{\exp\left(Y_{i,t-1}^{2}\right)}+\varepsilon_{i,t} \end{array}$$

where *c*_1_, *c*_2_, and *c*_1_ are threshold values; the initial values *Y*_*i*, −1_ = *Y*_*i*, 0_ = 0, ε_1,*t*_ = *c*_1_ if ε_*t*_ ≥ *c*_1_ and $\varepsilon_{1,t}=v_{t}^{\left(1\right)}$ if ε_*t*_ < *c*_1_, the initial stimulus variability $\varepsilon_{t}={\tilde{\varepsilon }}_{t}-\frac{1}{t}\overset{t}{\underset{i=1}{\sum }}{\tilde{\varepsilon }}_{i}$ ($\frac{1}{t}\overset{t}{\underset{i=1}{\sum }}{\tilde{\varepsilon }}_{i}\approx \omega_{t}$), ${\tilde{\varepsilon }}_{t}$ are the synaptic stimulus, and $v_{t}^{\left(1\right)}$ represent intrinsic noise and extrinsic or synaptic noise with small variance for *i* = 1, ··· , *m* and *t* = 1, 2, ··· , *n*. The encoder–decoder in Equations (8) and (9) describes a propagator of *n* signals through *m* relays.

The propagator in Equations (8) and (9) is also suitable for axons because an action potential that initiates in the axon causes back-propagating action potentials—a retrograde signal that travels in the opposite direction (Debanne, 2004) where α and β are the axial resistance and restoration coefficients.

#### 2.2.8. Conditions of Effective SSCCPI Circuit

The above analysis indicated that the precision, speed, and reliability of the SSCCPI circuit depended primarily on rate or temporal coding, rigorous, or near synchronous SSEs, the function of cortical columns, and the signal transfer precision in interneurons. These results led to the following inference:

*Inference 1*. The SSCCPI circuit is fast, reliable, and precise (or effective) if it satisfies the following conditions:

- SSE delivery is at least partial non-overlap of spike trains in interneurons (asynchronous SSEs);
- Cortical columns prevent both repeatedly triggering SSE delivery and incorrectly synaptic connections between adjacent columns; and
- The propagator in interneurons functions as a temporal complete fidelity and reliable information propagator.

The conditions (i) and (ii) are called the effective functions of SSEs and cortical columns, respectively.

### 2.3. Evidence for Effective SSCCPI Circuit

#### 2.3.1. SSCCPI Scheme

* The SSCCPI circuit follows the leading hypothesis that a synchronous firing chain is connected in a feed-forward manner where nerve impulses travel synchronously back and forth between layers; each neuron in a layer provides an excitatory connection to neurons in the next layer; and each neuron in the receiving layer is excited by neurons in the previous layer proposed by Abeles (1982a, 1991). An effective SSCCPI circuit supports the finding that the network topology of the feed-forward network determines the propagation of synchronous firing chain (Guo et al., 2017; see Han et al., 2015).

#### 2.3.2. Temporal Coding

* Experimental evidence supports temporal coding in the cortex (Abeles et al., 1993; Mainen and Sejnowski, 1995; de Ruyter van Steveninck et al., 1997; Nowak et al., 1997; Riehle et al., 1997; Frisina, 2001; Nemenman et al., 2008; Tiesinga et al., 2008). Most interneurons in subcortical areas utilize temporal coding in processing auditory information (Gao and Wehr, 2015), and temporal information within an acoustic signal is directly represented in the temporal patterns of neural activity throughout most parts of the auditory pathway leading to the auditory cortex (Wang et al., 2008).

#### 2.3.3. Asynchronous SSE Delivery

* High-intensity stimuli from external environment trigger SSEs: Japanese cartoons induce seizures in hundreds of children by intermittent photic stimulation (da Silva and Leal, 2017).
* High-intensity stimuli from task-related actions trigger SSEs: Elective attention and attention switching are fundamental to almost all cognitive tasks, which causes a substantial increase in stimulus intensity. Evidence for task-related SSEs includes that SSEs occur across neurons in the sensorimotor cortex (Murthy and Fetz, 1996) and the primary motor cortex of monkeys in relation to behavior (Riehle et al., 1997; Torre et al., 2016b); transient SSEs correlate not only with behavior but also with a mesoscopic brain signal, corroborating its relevance in cortical processing (Denker et al., 2011); the frequency of synchronous firing is modulated by behavioral performance and is specific for memorized visual stimuli (Pipa and Munk, 2011); neurons can synchronize their spiking in higher cortical areas when monkeys successfully solve visual recognition tasks (Gochin et al., 1994; Anderson et al., 2006) or process facial features (Hirabayashi and Miyashita, 2005); and most neuron pairs in a monkey's secondary somatosensory cortex fire synchronously in switching attention between two different tasks, and the degree of synchrony is affected by the attenuation state (Steinmetz et al., 2000; Roy et al., 2007).
* Response latency induces SSEs: Time delay enhances neural synchrony (Dhamala et al., 2004; Jirsa, 2008).
* Asynchronous SSEs are due primarily to synaptic noise: The uncertainty involved in the exact timing of neurotransmitter release causes synaptic noise even if repeated stimulation with identical stimulus evokes similar but not identical neuronal responses (Softky and Koch, 1992; Mainen and Sejnowski, 1995).
* Asynchronous SSEs relate to healthy neurons in the same areas of the brain fire (Fisher et al., 2005). In contrast, rigorous SSEs relate to brain disorders for Parkinson's disease (Rubchinsky et al., 2012), epilepsy (Jiruska et al., 2013), schizophrenia, autism, and Alzheimer's disease (Uhlhaas and Singer, 2006).
* The response latencies typically become shorter as the stimulus intensity increases (Henry and Lucas, 2008) but are greater at higher synaptic levels regardless of intensity (Klug et al., 2000); the increase in response latency produced by excess GMP is inversely proportional to the stimulus intensity (Nicol and Miller, 1978). The observation suggests the role of SSEs in shortening the wait times. From a commonsense point of view, the higher the signal intensity, the greater the spike density; thus, the waiting time to process the signal, and vice versa. Neuronal latencies can be as small as 0.1 ms and as large as 44 ms and the first spike latencies range roughly from 5 to 50 ms (Izhikevich, 2006).

Together the above observations support that asynchronous SSEs were usual while synchronous SSEs were unusual; asynchronous SSE delivery improved the transfer speed of high-intensity stimuli by shortening or avoiding the waiting time caused by response latencies.

#### 2.3.4. Parallel Propagation in Minicolumns

* Vertical columns are distributed in numerous cortical areas (Mountcastle, 1957; Buxhoeveden and Casanova, 2002; Opris and Casanova, 2014). Neurons within a minicolumn receive common inputs, have common outputs, and are interconnected (Cruz et al., 2005; Horton and Adams, 2005), which provides the possibility of decomposing the input spike trains and composing the output spike trains. The vibrissae on rodent snouts are topographically represented in the contralateral somatosensory cortex by distinct barrels in layer IV (Woolsey and van der Loos, 1970), which supports information flows in vertical columns starting from layer IV.
* Neurons within a given minicolumn are stereotypically interconnected in the vertical dimension (Rakic, 2008), which prevents repeated SSE delivery and thereby ensures signal parallel propagation within minicolumns.
* Columnar segregation (adjacent columns are segregated) is observed in the cat somatosensory cortex (Mountcastle et al., 1955), macaque somatosensory cortex (Powell and Mountcastle, 1959), and human extrastriate cortex (Horton and Adams, 2005; Tootell and Nasr, 2017). Columnar segregation stays functionally isolated by avoiding indiscriminate connections with local neurons and afferent axons there (Favorov et al., 2015), which prevents information loss and distortion caused by the decomposed propagation.
* The nervous system overwhelmingly prefers parallel computations over serial ones in time-critical applications; upward and downward connections within the thickness of the cortex are much denser than the connections that spread from side to side (Schrader et al., 2009), suggesting a columnar flow of information across layers as well as a laminar flow within some layers (Hawkins et al., 2017).
* The SSCCPI' signal parallel propagation in minicolumns is consistent with Mountcastle's cortical column hypothesis (1957). The latter requires that neurons in middle layers of the cortex, in which thalamic afferents terminate, should be joined by narrow vertical connections to cells in layers lying superficially and deep from them, so that all neurons in the column are excited by incoming stimuli with only small latency differences (Jones, 2000).

### 2.4. Evidence for Effective Encoder-Decoder

#### 2.4.1. Neural Basis of Multithreshold Decoder

* All-or-none modulation is a rule of a neuron's stimulus-response. Recent research shows that visual perception of simple stimuli is associated with an all-or-none cortical evoked response, the temporal precision of which varies as a function of perceptual strength (Sekar et al., 2012).
* Evidence suggests that active backpropagation facilitates the return of the attenuated stimulus to the original level by augmenting it with its previous excessive current: (i) An action potential that initiates in the cell body evokes a voltage spike to the axonal ending and then back through to the dendritic arbors; the basal, oblique, apical trunk; and tuft dendrites (Stuart and Sakmann, 1994; Waters et al., 2003) from which much of the original current originates. (ii) Backpropagation typically occurs only when the cell is activated to fire an action potential, and the extent of this backpropagation increases with the number and frequency of action potentials and depends on subthreshold excitatory inputs (Larkum et al., 1999), on the preceding rate of depolarization (Azouz and Gray, 2000, 2003) and on the preceding interspike intervals (Henze and Buzsáki, 2001; Badel et al., 2008).
* Graded potentials are on the same scale as the magnitude of stimuli (Purves et al., 2008) and subsequently influence transmembrane ion flow to either increase (excitatory) or decrease (inhibitory) the opportunity to fire. Effects of graded potentials are observed to be additive. Stimulus responses can be summed to increase the amplitude of graded potentials both spatially (multiple simultaneous inputs) and temporally (repeated inputs). Summation is the additive effect of multiple subthreshold graded postsynaptic potentials that determines whether the membrane potential will reach the threshold potential to generate an action potential. Hence, additive effects of graded potentials enable active backpropagation to facilitate the stimulus reconstruction.
* Threshold variation has been observed *in vivo* (Azouz and Gray, 1999; Henze and Buzsáki, 2001; Naundorf et al., 2006; McCormick et al., 2007; Yu et al., 2008), which provides evidence for multithreshold amplitude modulations. The multiple appropriate thresholds can be viewed as the result of evolution.

In summary, together all-or-none modulation, active backpropagation, additive graded potentials, and multithreshold amplitude modulations are neural evidence supporting the decoder in Equations (8) and (9). The all-or-none modulation and active backpropagation play the following key roles: (i) the neural response to stimulus at any strength above the threshold is the same; (ii) no action potential occurs if a neuron does not reach the threshold; and (iii) previous excessive currents compensate for the attenuated synaptic stimulus. Role (ii) prevents an over response that may cause signal distortion or incorrectly identify a noise as a signal. Role (iii) avoids an under response that may cause signal loss by receiving previous excessive current due to Role (i).

#### 2.4.2. Stable Fixed-Point Homeostatic Encoder

According to the current viewpoint, action potential encoding is implemented by transitions between a stable fixed point and a stable periodic cycle. The theoretical parameter intervals of the NLARI process are given by 0 < α < 2, 0 < β < 4, and 0 < γ < 1 for the stable fixed point and 0 < α < 2, $0<\beta <4\sqrt{e}$, and $1<\gamma <\sqrt{e}$ for the stable periodic cycle where γ = β/(4 − 2α) (for aperiodic cycles and chaos see He, 2018). Thus, we focused on identifying whether the NLARI's parameter values in Equation (4) lied alternately in the theoretical intervals of the stable fixed point and periodic cycle for real data by carrying out simulations. Independent evidence for precise spike timing in cortical neurons comes from intracellular recordings *in vitro*. Thus, we adopted intracellular recordings from the right parietal 4 (RP4) neuron of a snail elicited by the application of paeonol as the received stimuli entering the encoder. The recordings were made by the method described by Chen et al. (2010).

Surprisingly, our statistical results indicated that the dynamic mechanism of action potential encoding was a single stable fixed point, but not transitions between a stable fixed point and a stable periodic cycle or a single stable periodic cycle. This is because all the confidence intervals of these parameters lied inside the theoretical parameter intervals for a single stable fixed point at the 99% confidence level for all the recordings. This result could be viewed as a consequence of ionic homeostatic regulation for maintaining the resting potential. Whether the stability coefficient lies in (0, 1) or $\left(1,\sqrt{e}\right)$ crucially determines whether the encoding dynamic mechanism is the stable fixed point or the stable periodic cycle. The stability coefficient comprises the membrane resistance coefficient and the membrane restoration coefficient. The permeability of ionic channels causes the membrane resistance, preventing rapid changes in the membrane potential. The electrochemical driving force restores the changed membrane potential into the resting potential by driving Na^+^ and K^+^ ion influx/efflux across the membrane and synaptic plasticity of strengthening/weakening between neighboring synapses in response to increases and decreases in their activity. Homeostasis is the most basic way the body maintains a stable internal environment. Ionic homeostasis supports a stable fixed-point encoder by regulating changes to synaptic size, synaptic strength, and ion channel function in the membrane (for recent study see Davis and Bezprozvanny, 2001).

**Figure 8:**
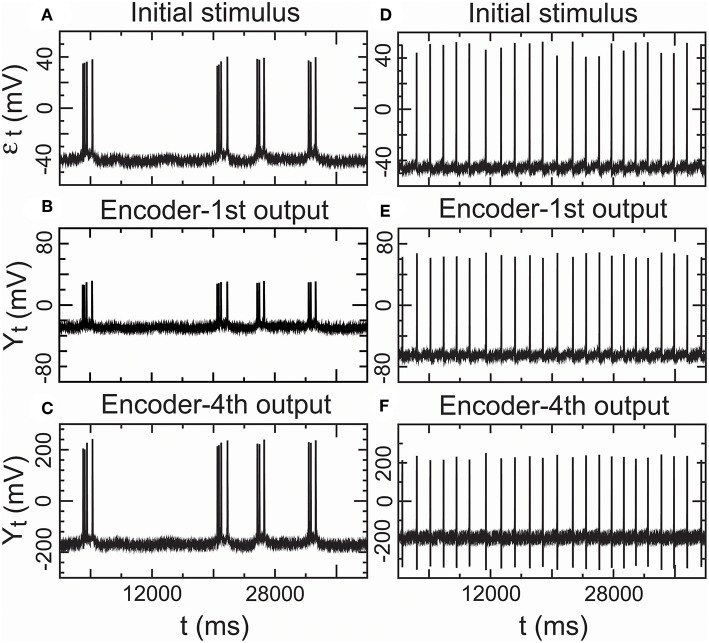
Performance of the encoder in nomodulation using real low-density inputs. **(A)** Initial stimulus: membrane potentials recorded 20 min after the administration of paeonol. **(B)** First encoder output driven by the initial stimulus **(A)**. **(C)** Fourth encoder output based on the initial stimulus **(A)**. **(D)** Initial stimulus: spontaneous action potentials (no paeonol). **(E)** First encoder output driven by the initial stimulus **(D)**. **(F)** Fourth encoder output based on the initial stimulus **(D)**. These results showed that the encoder could exactly encode stimulus input with low density in a simple neural circuit with a few interneurons.

Furthermore, we assessed the performance of the encoder as a propagator with an encoder in Equation (7) in nomodulation by the degree of consistency between the input and output spike trains of the encoder. Figure 8A presents intracellular recordings from the right parietal 4 (RP4) neuron of a snail elicited by the application of paeonol. Figure 8D presents intracellular recordings from the spontaneous action potentials (no paeonol) for the same neuron. All of the first outputs (*Y*_1,*t*_) of the encoder in Figures 8B,E and the fourth outputs (*Y*_4,*t*_) of the encoder in Figures 8C,F were consistent with their received stimuli. This result showed that neuronal responses to background stimuli resembled the stimuli and that the encoder exactly predicted information transfer through a few relays. Additionally, we again observed that nerve impulses were significantly enlarged in the firing as shown in Figure 7, which reflected the phenomenon that the opening of voltage-gated channels trends to elicit significantly larger currents than the original stimulus.

#### 2.4.3. Temporal Precision in Real Low Density Inputs

We proved that the encoder-decoder in Equations (8) and (9) was a complete fidelity information propagator as an important condition for an effective SSCCPI circuit. The propagation success rates of the two real input spike trains through 3,000 interneurons could reach 100.00% for the lower density spike train (the application of paeonol) and 99.85% for the higher density spike train (no paeonol). These results satisfied the condition that the encoder-decoder was a complete fidelity information propagator.

**Figure 9:**
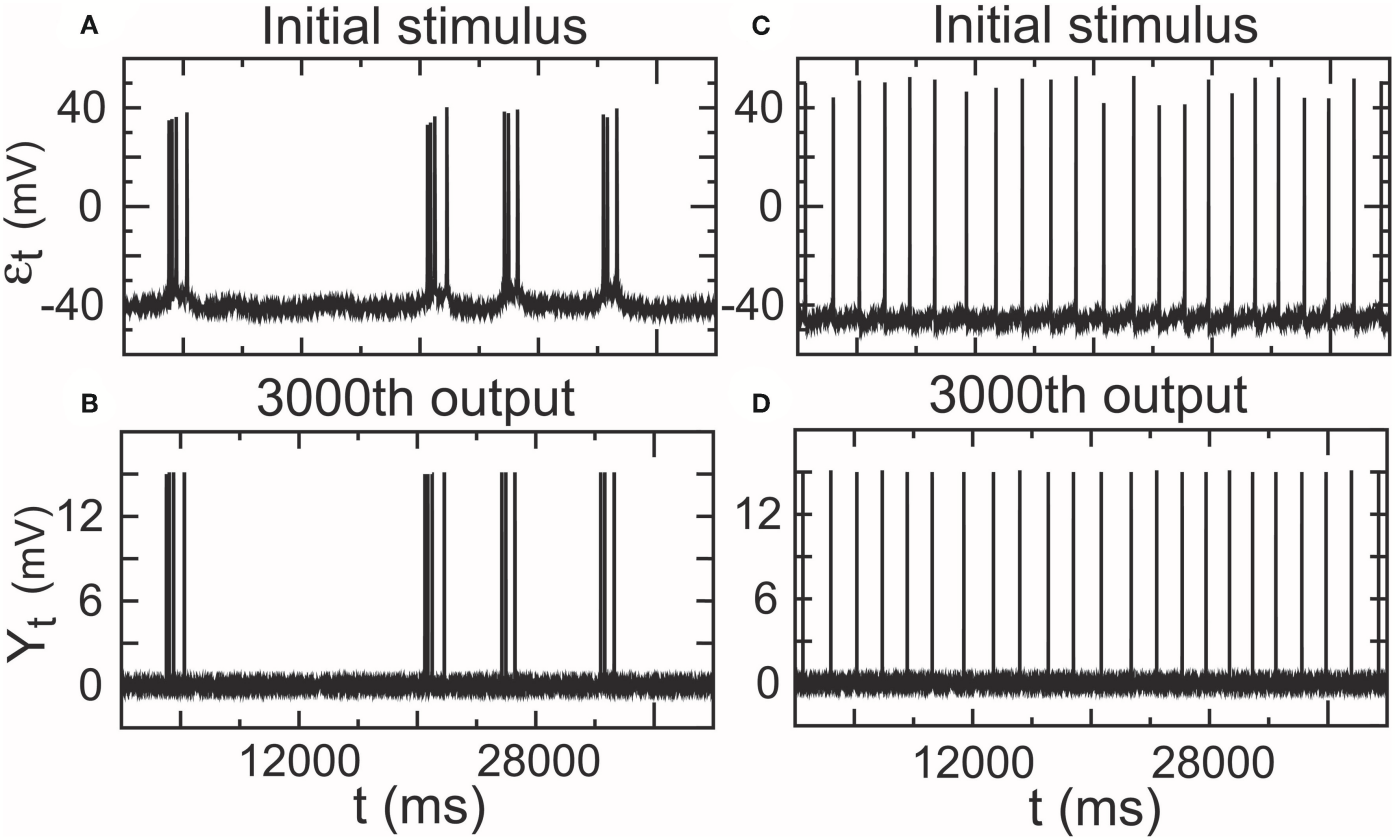
Performance of the encoder–decoder in all-or-none modulation using real low-density inputs. **(A)** Initial stimulus: membrane potentials recorded 20 min after the administration of paeonol. **(B)** 3,000th output of the encoder–decoder based on the initial stimulus **(A)**. **(C)** Initial stimulus: spontaneous action potentials (no paeonol). **(D)** 3,000th output of the encoder–decoder based on the initial stimulus **(C)**. These results showed that the encoder–decoder could exactly encode stimulus input with low density in a complex cortical circuit with many interneurons.

Furthermore, we showed that the encoder-decoder in all-or-none modulation gave a good performance that simulated information transfer through 3,000 relays by repeatedly operating the propagator for *m* = 3, 000 times initiated by the two real spike trains mentioned above. The outputs (*Y*_3,000,*t*_) (Figures 9C,D) for the propagator generated by Equations (8) and (9) exhibited consistent trajectories with the initial stimulus (ε_*t*_) (Figures 9A,B).

#### 2.4.4. Temporal Precision in Fitting High Density Inputs

**Figure 10:**
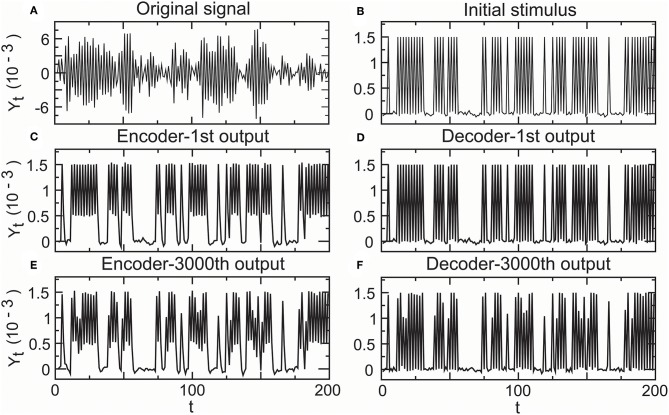
Performance of the encoder–decoder in simulated high-density inputs. **(A)** Original signal: a realization of a sound wave simulator. **(B)** Initial stimulus received by all-or-none modulation. **(C)** First encoder output driven by the initial stimulus. **(D)** First decoder output for correcting the error of the first encoder output. **(E)** 3,000th encoder output based on the initial stimulus. **(F)** 3,000th decoder output for correcting the error of the 3,000th encoder output.

In the above simulation studies, we adopted low density stimuli as an input spike train. Recent research indicates that the primary auditory cortex uses a temporal representation to encode slowly varying acoustic signals and a firing rate-based representation to encode rapidly changing acoustic signals (Wang et al., 2008). For this reason, we need to investigate whether the temporal encoder–decoder in Equations (8) and (9) is still a complete fidelity information propagator initialized by high density stimuli. In addition, it is not clear how the decoder in Equation (8) corrects the error of the encoder in Equation (9). Hence, we carried the following simulation. Let a random sound generator produce a spike train with high density spike trains as an original signal input (Figure 10A). The fitting initial stimulus input was received by all-or-none modulation (ε_1,*t*_) (Figure 10B). Although the first output of the encoder (*Y*_1,*t*_) somewhat deviated from the initial stimulus (Figure 10C), the deviation was removed by the decoder. In the first output of the decoder, the signal had already returned to its initial state (ε_2,*t*_) (Figure 10D). The 3, 000th output of the encoder (*Y*_3000,*t*_) deviated (Figure 10E), but the decoder corrected this deviation (ε_3000,*t*_) (Figure 10F). The simulation result proved that the encoder–decoder retained good performances at high firing rates.

#### 2.4.5. Temporal Reliability of Encoder-Decoder

According to our analysis, the SSCCPI transfer reliability relies on the reliability of single-neuron (or interneuron) transfer precision if SSE delivery and cortical columns function normally. Thus, we focused on identifying whether the single-neuron transfer precision was influenced by irregular stimuli. To address this issue, we investigated whether there was distinct difference between the precision of one transmission and the average precision of numerous transmissions.

The cerebral cortex typically consists of the six-layered neocortex. If each layer contains at least one interneuron, then the number of interneurons through which signal is transmitted is at least six. For this reason, we considered the number of relays to be 6 and 3000. Each spike train comprised 200 signal points. The original stimuli were generated by the realization of a random sound generator by Equation (4) where the stimuli represented a rapidly changing signal but not a constant stimulus. We simulated the transfer of spike trains driven by one firing and 3,000 firings by repeatedly operating the propagator in Equations (8) and (9) once and 3, 000 times. We calculated the propagation success rates of one transmission and the average propagation success rates of 10,000 transmissions across the stable fixed-point range by increasing the stability coefficient value from 0 to 1 based on Equations (2) and (3).

**Figure 11:**
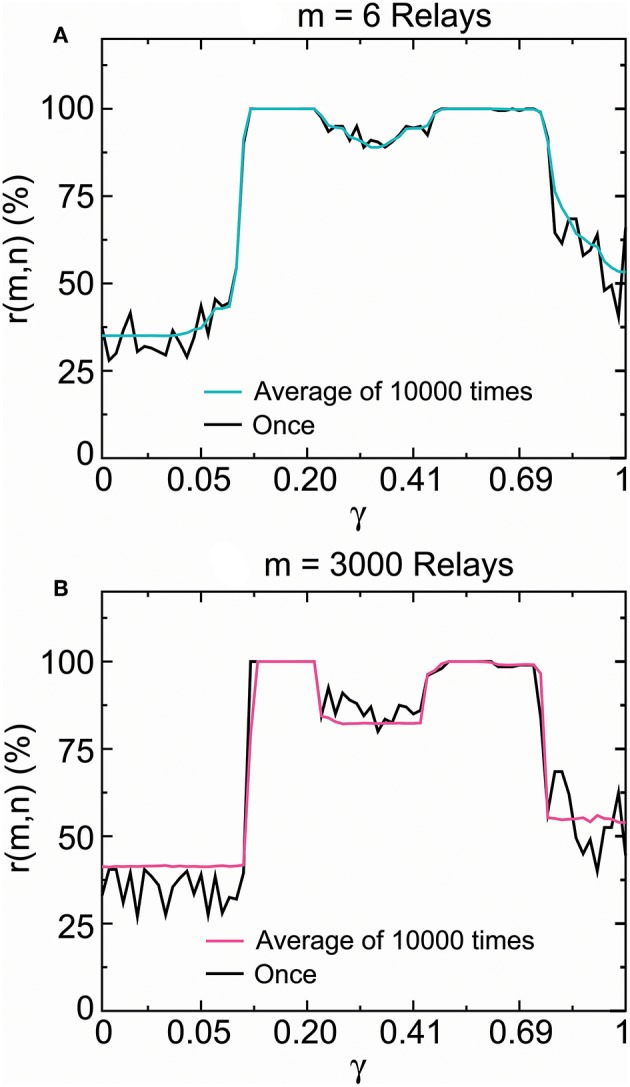
SSCCPI transfer reliability in simulated high-density inputs. Comparison of the propagation success rate of one realization with that of the average over 10,000 realizations by running the encoder–decoder 6 times (6 relays) **(A)** and 3,000 times (3,000 relays) **(B)** where the signal number is 200. These results indicated that SSCCPI transfer precision remained reliable under the irregular firing condition if relay single-neuron transfer was completely successful in a temporally precise manner.

Figure 11 presented the distribution of these propagation success rates in the stable fixed-point range. The propagation success rate was above 99.982% in 0.118 < γ < 0.209 and 99.974% in 0.500 < γ < 0.613 for six relays (Figure 11A), and above 99.986% in 0.118 < γ < 0.209 and 99.982% in 0.500 < γ < 0.575 for 3,000 relays (Figure 11B). In contrast, the propagation success rates were under 89% outside the interval 0.099 < γ < 0.797 for six relays (Figure 11A) and 82% outside the interval 0.11 < γ < 0.77 for three-thousand relays (Figure 11B). According to the results obtained in section 2.2.2, the SSCCPI propagation success rate achieved above 97% for 80 and 74% for 1,000 receiving neurons inside the intervals 0.118 < γ < 0.209 and 0.500 < γ < 0.575 where the single-neuron propagation success rate was above 99.97%, but was almost zero for 80 receiving neurons outside the interval 0.0997 < γ < 0.798 where the single-neuron propagation success rate was under 89%.

From Figure 11 we surprisingly discovered that there was no distinct difference between the single-neuron transfer precision of one firing and the average transfer precision of many firings in the stable fixed-point range. In particular, there was almost no difference in the complete success propagation interval. This result suggested that the influence of firing irregularity on the transfer precision was not distinct, especially for complete temporal fidelity transfer. We see that an appropriately large stability coefficient played the key role in the achievement of high transfer precision and reliability in single neurons, while the effects of firing irregularity and relay number were not distinct. The ionic homeostasis regulating the stability coefficient into an appropriate stable fixed-point range for the complete propagation success could be regarded as a result of long term evolution.

Our simulation results indicated that the encoder-decoder could be a complete fidelity and reliable information propagator of temporal information in certain specific stable fixed-point interval. Experimental evidence supports temporal precision with millisecond fidelity and reliability (Abeles et al., 1993; Mainen and Sejnowski, 1995; de Ruyter van Steveninck et al., 1997; Ikegaya et al., 2004; Gollisch and Meister, 2008; Nemenman et al., 2008; Tiesinga et al., 2008).

## 3. Discussion

In this study, we proposed the SSCCPI circuit in a cortical network model for cortical mechanisms of high intensity signal transfer over a background of irregular firing and response latency. We hypothesized that a thalamic high-intensity stimulus input triggered SSEs, and dense spikes were scattered to many receiving neurons within a cortical column in layer IV. Then, many sparse spike trains from the receiving neurons as signals for reversible disassembly were propagated in parallel by the propagator (encoder–decoder) in interneurons along minicolumns through layers II/III with less latency and finally integrated into an output spike train toward or in layer Va. The encoder in interneuron of minicolumns was derived by modeling the membrane potential in response to stimulus as the input and output in a stochastic resilience system using the NLARI process. The multithreshold decoder was introduced to correct encoding errors. We derived the conditions for an effective (fast, reliable, and precise) SSCCPI circuit: SSEs were asynchronous (near synchronous or at least partial non-overlap); critical columns had the capability to prevent both repeated SSE deliveries and incorrect synaptic connections between adjacent columns; and the encoder-decoder in interneurons was a temporal complete fidelity and reliable information propagator. There is evidence supporting the effective transfer functions of SSEs and cortical columns. An increasing body of real evidence suggests that the neuronal coding could be a temporal fidelity and reliable information propagator. Our simulations demonstrated that the encoder-decoder could be temporal complete fidelity and reliable in certain special intervals contained within the stable fixed-point range. Moreover, the encoder-decoder simulated the mechanism by which incoming and outgoing impulses of each neuron remain temporally equational each time by achieving the response error correction at the next fire command. This result explained why the influence of relay number on the signal propagation precision was not distinct.

Our findings also include the following: (i) The transfer speed in the SSCCPI circuit depended crucially on the degree of non-overlap of SSE delivery: the higher the non-overlap, the faster the transfer speed, which reflected the key role of synaptic noise in improving the signal transfer speed. (ii) When SSEs and cortical columns have effective transfer functions, the SSCCPI' reliability depended on the reliability of the single-neuron propagator. (iii) A temporal complete fidelity propagator was reliable and the effect of firing irregularity on the single-neuron transfer precision was not distinct. (iv) Substantially increased output firing rates resulted from rigorous SSEs plus the interneuron-transfer mistake in a shift in spike timing or the rise of a spike, while any change in the output spike train was unlikely to result from asynchronous SSEs plus any single-neuron transfer mistakes. (v) Asynchronous SSEs were a cortical population response to high-intensity thalamic inputs, whereas rigorous SSEs might be viewed as a cortical population response to ultrahigh-intensity thalamic inputs or neural damage that significantly reduced the limiting ability of individual neurons to process information. (v) The dynamic mechanism of action potential encoding was a single stable fixed point, which was attributed to ionic homeostasis, but not transitions between a fixed point and a limit cycle. (vi) All-or-none modulation prevented an over response but failed to correct an under response. (vii) Backpropagation corrected an under response. (viii) There has been a long-standing debate about the function of SSEs (Abeles, 1982b; Shadlen and Movshon, 1999; Singer, 1999; Pipa and Munk, 2011). Cortical columns are thought to have a structure without a function (Horton and Adams, 2005). Here we hypothesized that a high-intensity thalamic input triggers SSEs. Moreover, we hypothesized that cortical columns prevented repeatedly triggering SSE delivery and fast parallel propagation within minicolumns and information loss caused by the disassembly propagation.

The present results suggest that any neural alterations in the SSCCPI circuit possibly cause brain disorders and thereby may give an insight into the exact etiologies of neurocognitive disorders. For example, according to our analysis, rigorous SSEs plus a single-neuron transfer mistake may induce substantially increased output firing rates as seen in an epileptic seizure; the breakdown of columnar segregation may destroy information during disassembly-parallel propagation through one layer to the next, which may cause cognitive disease. Additionally, this study introduced the membrane potential waveform indicators to assess the influence of synaptic stimulation input on the membrane potential. Together with the wave indicators, the SSCCPI circuit may be applied to the signal processing pathways in cognitive tasks. We expect that these issues will attract more attention and intensive research.

## 4. Methods

### 4.1. Datasets

The real spike trains were intracellular recordings from the right parietal 4 (RP4) neuron of a snail elicited by the application of paeonol (the dataset and programs are presented in Supplementary Table 1). Neuronal recordings were obtained with the method described by Chen et al. (2010).

### 4.2. Statistical Method

#### 4.2.1. Estimations of the Waveform Indicators for (Figure 6)

Let △*Y*_*t*_ = *Y*_*t*_ − *Y*_*t*−1_. Equation (4) can be rewritten as:

(10) $$\begin{array}{l} \Delta Y_{t}=\theta_{1}\Delta Y_{t-1}+\theta_{2}\frac{-Y_{t-1}}{e^{Y_{t-1}^{2}}}+\varepsilon_{t} \end{array}$$

Note that *Y*_*t*_ = *X*_*t*_ − *X*_0_ − (ω/α)*t* in Equation (10). Consider the regression line *X*_*t*_ = *a* + *bt* + *u*_*t*_ where *a* = *X*_0_ and *b* = ω/α. We obtained the estimates â and $\hat{b}=\hat{\omega }/\hat{\alpha }$ by estimating the regression line using the ordinary least squares (OLS) method with real data {*X*_*t*_}. We got the OLS estimates ${\hat{\theta }}_{2}=\hat{\beta }$ and $\hat{\sigma }$ by estimating (Equation 10) using data {*Y*_*t*_} where $Y_{t}=X_{t}-â-\hat{b}t$. Then, the slope indicator and amplitude indicators were given by $\eta_{1}=\hat{b}$ and $\eta_{2}=\hat{\sigma }/\hat{\beta }$.

#### 4.2.2. Parameter Estimations for (Figures 7–9)

The NLARI's stable fixed point is exponentially asymptotically stable but not globally stable (He, 2013), which implies that a large stimulus may trigger a poor response. Decreasing the absolute values of the data can usually prevent this problem (He, 2014). Therefore, to make good estimates, we first performed data preprocessing by letting *Y*_*t*_ = real data/1, 000 where *Y*_*t*_ were real data or simulated data generated by Equation (4). Thus, we estimated (Equation 10) and obtained the estimates $\hat{\alpha }=1-{\hat{\theta }}_{1}$, $\hat{\beta }={\hat{\theta }}_{2}$, and $\hat{\gamma }=\hat{\beta }/\left(4-2\hat{\alpha }\right)$.

#### 4.2.3. Testing for the Stable Fixed-Point Encoder in Equation (4)

For the NLARI's stable fixed-point range, the theoretical intervals of the parameters α, β, and γ are given by (−1, 1), (0, 4), and (0, 1), respectively (for more detailed information see He, 2014). The confidence intervals of these parameters for large samples are based on the standard normal distribution. When the γ value is significantly greater than zero, the hypothesis tests whether real data are generated by the NLARI process in the stable fixed-point range can be achieved by a confidence interval approach for the standard normal distribution. Therefore, in this case, we only need to perform a test to determine whether the confidence intervals ${\hat{\theta }}_{1}\pm z_{0}s\sqrt{s^{11}}$ for α, ${\hat{\theta }}_{2}\pm z_{0}s\sqrt{s^{22}}$ for β, and $\hat{\gamma }\pm z_{0}{\hat{\sigma }}_{\hat{\gamma }}$ for γ lie in the intervals (−1, 1), (0, 4), and (0, 1), respectively, where *z*_0_ represents a critical value at a common significance level for the *t* distribution (e.g., the critical value of 1.645 is significant with ∞ at the 0.05 level in right-hand-tail). Our results based on the OLS estimates of Equation (10) indicated that all the parameter estimations based on the recordings used in this study fell significantly inside the theoretical intervals for the stable fixed-point range.

### 4.3. Simulation Method

We calculated the outputs of the propagator in Equation (7) initiated by real neuronal data for Figures 7, 8, in Equations (8) and (9) initiated by real neuronal data for Figure 9, in Equations (8) and (9) initiated by a random stimulator for Figure 10, and the propagation success rate based on Equations (1) and (2) by repeatedly running the propagator in Equations (8) and (9) initiated by a random stimulator for Figure 11.

#### 4.3.1. Calculations for (Figures 7–9)

The calculation results were obtained by performing the following steps:

**Step 1**. Initial values: Raw data set contains 20, 000 points from 1 to 40, 000 ms in increments of 2 ms. The initial stimuli were given by ${\tilde{\varepsilon }}_{t}=$ real data/1, 000 in Figures 7, 8 and $\varepsilon_{t}={\tilde{\varepsilon }}_{t}-\frac{1}{t}\overset{t}{\underset{i=1}{\sum }}{\tilde{\varepsilon }}_{i}$ in Figure 9 where the real data were recordings for paeonol at a concentration of ≥ 1.2 mmol/L in Figures 7A–9A and recordings for no paeonol in Figures 8D, 9C. Select the parameters of the encoder γ ∈ (0.27, 0.41), α ∈ (0, 2), and β = γ(4 − 2α) (e.g., α = 0.71, β = 0.7, γ = 0.2713) and the parameters of the decoder *c*_1_ = 0.0015, *c*_2_ = 0.0010, *c*_3_ = 0.0008, and $\sigma_{1}=2^{-6}$. Let *n* = 600 and *m* = 3, 10, 17, 18 in Figure 7, *n* = 20, 000 and *m* = 1, 4 in Figure 8, and *n* = 20, 000 and *m* = 3, 000 in Figure 9.

**Step 2**. Encoder: Produce the outputs of the encoder *Y*_*i,t*_ in Equation (7) in nomodulation in Figures 7B, 8B,C,E,F, the encoder *Y*_*i,t*_ in Equation (7) in all-or-none modulation by ε_1,*t*_ = *c*_1_ if ε_*t*_ ≥ *c*_1_, $\varepsilon_{1,t}=v_{t}^{\left(1\right)}$ if ε_*t*_ < *c*_1_, and *Y*_*i* −1, *t*_ = *c*_1_ if *Y*_*i* −1, *t*_ ≥ *c*_1_ and $Y_{i\;-1,t}=v_{t}^{\left(1\right)}$ if *Y*_*i* −1, *t*_ < *c*_1_ for *i* ≥ 2 in Figure 7C.

**Step 3**. Encoder-Decoder: Produce the outputs of the encoder *Y*_*i,t*_ in Equation (9) and the decoder value ε_*i,t*_ were given by Equation (8) in Figures 9B,D.

**Step 4**. Outputs: The final outputs of the encoder and the decoder were given by ${Ŷ}_{i,t}=10^{5}\times Y_{i,t}$ and ${\hat{\varepsilon }}_{i,t}=10^{5}\times \varepsilon_{i,t}$ where *t* = 1, ··· , *n* and *i* = 1, ··· , *m*.

#### 4.3.2. Calculations for (Figures 10, 11)

The simulation results were obtained by performing the following steps:

**Step 1**. Initial values: Produce the initial stimulus ε_*t*_ by ε_*t*_ = *c*_1_ if *Y*_*t*_ ≥ *c*_1_ and $\varepsilon_{t}=v_{t}^{\left(1\right)}$ if *Y*_*t*_ < *c*_1_ where the original signal *Y*_*t*_ was generated by Equation (4) based on α = 0.71, β = 0.70, γ = 0.97, σ = 0.0011, and Gaussian white noise $v_{t}^{\left(1\right)}\sim i.i.d.N\left(0,\sigma_{1}^{2}\right)$ with $\sigma_{1}=2.7\times 10^{-5}$ for *n* = 200.

**Step 2**. Encoder-Decoder: Produce the output of the encoder *Y*_*i,t*_ in Equation (9) based on α = 1 − 0.005*j*, γ = 0.0133*j*, and β = γ(4 − 2α) for *j* = 25 (i.e., α = 0.8750, β = 0.7481, and γ = 0.3325) and the decoder ε_*i,t*_ in Equation (8) driven by the initial stimulus ε_*t*_ based on *c*_1_ = 0.0015, *c*_2_ = 0.0010, *c*_3_ = 0.0008 for *n* = 200 and *m* = 1, 100, 3, 000.

**Step 3**. Success Rate: Calculate the propagation success rate *r*(*m, n*)_*j*_ by Equation (1) where the initial input **v**_0_ and the final output **v**_*m*_ are defined by (Equation 2).

**Step 4**. Average Success Rate: Repeat Steps 1 to 3 for *T* = 10, 000 to calculate $r\left(m,n\right)=\frac{1}{T}\overset{T}{\underset{j=1}{\sum }}r{\left(m,n\right)}_{j}$.

**Step 5**. Average Success Rate Distribution: Repeat Steps 1 to 4 for γ = 0.0133*k* within (0, 1), α = 1 − 0.005*k*, β = γ(4 − 2α) for *k* = *k* + 1 from *k* = 1 to *k* = 75.

## Data Availability

All datasets generated for this study are included in the manuscript and the Supplementary Files.

## Author Contributions

The author confirms being the sole contributor of this work and has approved it for publication.

### Conflict of Interest Statement

The author declares that the research was conducted in the absence of any commercial or financial relationships that could be construed as a potential conflict of interest.

## Supplementary Material

Supplementary Table 1 Real dataset and programs.
